# Isoprostanes and 4-Hydroxy-2-nonenal: Markers or Mediators of Disease? Focus on Rett Syndrome as a Model of Autism Spectrum Disorder

**DOI:** 10.1155/2013/343824

**Published:** 2013-06-13

**Authors:** Cinzia Signorini, Claudio De Felice, Thierry Durand, Camille Oger, Jean-Marie Galano, Silvia Leoncini, Alessandra Pecorelli, Giuseppe Valacchi, Lucia Ciccoli, Joussef Hayek

**Affiliations:** ^1^Department of Molecular and Developmental Medicine, University of Siena, I-53100 Siena, Italy; ^2^University General Hospital, Neonatal Intensive Care Unit, Azienda Ospedaliera Universitaria Senese, I-53100 Siena, Italy; ^3^Institut des Biomolécules Max Mousseron (IBMM), UMR 5247-CNRS-UM I-UM II, BP 14491 34093, Montpellier, Cedex 5, France; ^4^University General Hospital, Child Neuropsychiatry Unit, Azienda Ospedaliera Universitaria Senese, I-53100 Siena, Italy; ^5^Department of Life Science and Biotechnologies, University of Ferrara, I-44121 Ferrara, Italy; ^6^Department of Food and Nutrition, Kyung Hee University, Seoul 130-701, Republic of Korea

## Abstract

Lipid peroxidation, a process known to induce oxidative damage to key cellular components, has been implicated in several diseases. Following three decades of explorations mainly on *in vitro* models reproducible in the laboratories, lipid peroxidation has become increasingly relevant for the interpretation of a wide range of pathophysiological mechanisms in the clinical setting. This cumulative effort has led to the identification of several lipid peroxidation end-products meeting the needs of the *in vivo* evaluation. Among these different molecules, isoprostanes and 4-hydroxy-2-nonenal protein adducts appear to be particularly interesting. This review shows how specific oxidation products, deriving from polyunsaturated fatty acids precursors, are strictly related to the clinical manifestations and the natural history of Rett syndrome, a genetically determined neurodevelopmental pathology, currently classified among the autism spectrum disorders. In our experience, Rett syndrome offers a unique setting for physicians, biologists, and chemists to explore the borders of the lipid mediators concept.

## 1. Introduction

Oxidative stress (O.S.), a biological condition determined by the imbalance between prooxidant and the antioxidant system, is involved in several conditions, including inflammation, carcinogenesis, neurodegeneration, and development. Lipid peroxidation is a critical component of O.S. In particular, free radicals and specifically reactive oxygen species (ROS) are able to attack polyunsaturated fatty acids (PUFAs) of cell membranes thus generating a family of *α*,*β*-unsaturated reactive aldehydes, such as 4-hydroxy-2-nonenal (4-HNE) and prostaglandin-like end-products termed isoprostanes (IsoPs). Emerging knowledge points out the key role of these molecules in generating oxidative-driven damage. Therefore, these compounds, rather than being considered simple biomarkers of disease, are to be considered as mediators. In the present review, we discuss the evidence on the issue by focusing our proof-of-concept reasoning on the unique O.S. model of disease represented by Rett syndrome (RTT), a genetically determined autism spectrum disorder.

## 2. Role of Oxidative Stress in Human Diseases

O.S., as defined as an imbalance between cellular antioxidant defenses and free radicals production, is implicated in a wide variety of disease processes. To this regard, a role of oxidative damage in cancer [1, 2], neurodegenerative diseases [3–6], and likely inflammatory bowel disease [7, 8] has been recently reported. In the matter of atherosclerosis, an oxidative damage, especially to lipids, is certain. However, as it concerns the size of its effects and underlying pathogenetic mechanisms there is likely much less certainty than a few years ago [9]. One method to quantify oxidative injury is to measure lipid peroxidation. However, a cause-effect relationship is often difficult, if not impossible, to prove. 

## 3. Oxidative Stress: Shifting from the *In Vitro* to the *In Vivo* Concept 

Until the ‘80s, lipid peroxidation was confined to specialized laboratories in which the phenomenon was mainly evaluated *in vitro.* At this stage, researchers were interested in reproducing lipid peroxidation as a macrophenomenon induced by xenobiotics of such an entity that would be quite unlikely to be present in human physiology and pathology. The meaning of those experiments and tests was mainly to evidence the phenomenon by measuring the products generated in the process. In this particular setting, oxidized aldehydes (chiefly malondialdehyde, MDA, and all the thiobarbituric acid reactive substances, TBARS) were considered to be reliable indicators of lipid peroxidation. Things changed dramatically when a number of laboratories around the world evidenced increased levels of lipid peroxidation markers in tissues and body fluids from patients as compared to those observed in healthy controls. Thus, the setting has progressively moved from *in vitro* to *in vivo* and has allowed to enlighten the role of lipid peroxidation within the mechanisms of several diseases and physiologic signaling pathways.

The entity of the phenomenon became dramatically downscaled so that more sensitive and reliable markers *in vivo* became an urgent necessity. At this further stage, several key markers of lipid peroxidation applicable *in vivo* have been discovered, including IsoPs and 4-HNE protein adducts (4-HNE PAs). The measurement of those markers has allowed a much deeper understanding of the key role of oxidative stress in health and disease. Likely, we are now at the stage in which we are asking whether these end-peroxidation products may have biological activity on their own, thus progressively shifting from markers to mediators.

## 4. Lipid Peroxidation End-Products: Shifting from Markers to Mediators

Lipid mediators are chemical messengers that are released in response to tissue injury and include prostaglandins, leukotrienes, lipoxins, and neuroprotectins, playing an essential role in the different phases of inflammation. While early in the inflammatory response, arachidonic acid (AA) metabolites (i.e., prostaglandins and leukotrienes) exert proinflammatory actions, by promoting chemotaxis of phagocytic leukocytes and inducing fever; the so-termed “lipid mediator class switching” is responsible for the production of specialized proresolving mediators, such as lipoxins, resolvins, maresins, and protectins, which exert specific roles in counterregulating inflammation and turning on resolution [10, 11]. Thus inflammation can be considered a self-limited process depending on lipid-derived mediators produced in the inflammatory exudates.

Emerging evidence indicates that biomarkers of lipid peroxidation can be interpreted as lipid mediators, acting as key factors in the regulation of the delicate balance between inflammation and the resolution process. One unanswered major question is concerning whether the end-products of peroxidation, such as IsoPs, are to be considered true markers or simple markers of O.S.; in particular whether different lipid mediators would determine different diseases; or whether the time course of production is a critical factor in physiology and disease. 

## 5. Aldehyde Products

As mentioned earlier, the interaction of ROS and lipids can induce the peroxidation process, a chain reaction that produces multiple breakdown molecules, including many highly reactive aldehydes such as malondialdehyde, 4-HNE, 4-hydroxy-2-hexenal, and acrolein [12].

Among them, 4-HNE, a 4-hydroxyalkenal, is the most intensively studied aldehyde and it is one of the best recognized and most studied cytotoxic products derived from the lipid peroxidation processes. 

4-HNE derives from the oxidation of *ω*-6 PUFAs, essentially arachidonic and linoleic acid, that is, the two most represented fatty acids in biomembranes. 4-HNE is an unusual compound containing three functional groups that in many cases act in concert explaining its high reactivity. There is, first of all, a conjugated system consisting of a C=C double bond and a C=O carbonyl group in 4-HNE. The hydroxyl group at carbon four contributes to the reactivity both by polarizing the C=C bond and by facilitating internal cyclisation reactions, such as thioacetal formation [13].

Because of its chemical properties, 4-HNE is an amphiphilic molecule (water soluble while exhibiting strong lipophilic properties). 4-HNE tends to concentrate in biomembranes, where phospholipids, like phosphatidylethanolamine, and proteins, such as transporters, ion channels and receptors, quickly react with it. In addition, since it is a highly electrophilic molecule, it easily reacts with low molecular weight compounds, such as glutathione, and at higher concentration with DNA [12]. Due to its electrophilic nature, 4-HNE can form adducts with cellular protein nucleophiles. Indeed, the reactivity of 4-HNE explains its potential involvement in the modulation of enzymes activity, signal transduction, and gene expression [13].

Adduction to and modification of functional and/or signaling proteins most likely represents one of the main mechanisms by which 4-HNE and also the other *α*,*β*-unsaturated aldehydes can influence physiological as well as pathological processes. 

Primary reactants for 4-HNE are the amino acids cysteine, histidine, and lysine, which—either free or protein-bound—undergo readily Michael addition reactions to the C=C double bond. Besides this type of reaction, a secondary reaction may occur involving the carbonyl and the hydroxyl groups to form a cyclic hemiacetal derivative. Amino groups (e.g., Lys) may alternatively react with the carbonyl group of 4-HNE to yield a Schiff base product [14].

Therefore, since proteins play an important role in normal structure and function of the cells, oxidative modifications by increased 4-HNE levels, as in O.S. conditions, may greatly alter their structure. These protein alterations may subsequently lead to loss of normal physiological cell functions and/or may lead to abnormal function of cell and eventually to cell death. 

4-HNE adducts contribute to the pool of damaged enzymes, which increases in levels during aging and in several pathological states [15]. Furthermore, impaired protein clearance (i.e., ubiquitin proteosome system dysfunction) and/or the overwhelming production of abnormal proteins play an important role in the pathophysiology of disorders related to O.S., and a lot has been done in the last few decades on their role in neuro-related pathologies [16]. 

Based on this, 4-HNE represents one of the most useful biomarkers for the occurrence and/or the extent of O.S. [17].

## 6. Isoprostanes

IsoPs are a unique series of prostaglandin-like compounds generated, via a free radical-catalyzed mechanism, from a number of different PUFAs, including AA, eicosapentaenoic acid (EPA), adrenic acid (AdA), and docosahexaenoic acid (DHA).

Looking at the lipid composition of the tissues of the body [18], AA was found to be localized quite everywhere, whereas PUFAs such as DHA or AdA are mainly localized in nervous tissue and especially in grey and white matters. Thus the clinical relevance of the different classes of IsoPs hinges on the PUFA precursor anatomical distribution.

### 6.1. F_2_-Isoprostanes

The discovery of prostaglandin F_2_-like compounds, termed F_2_-isoprostanes (F_2_-IsoPs), generated by free radical-induced peroxidation of arachidonic acid, was for the first time reported by Morrow et al. [19].

Since F_2_-IsoPs, initially formed *in situ* on phospholipids [20], are released into the circulation and because these prostanoids are less reactive than other lipid peroxidation products (i.e., lipoperoxides and aldehydes), they can be detected more easily in plasma. Since the discovery of these molecules, F_2_-IsoPs have become the biomarker of choice for assessing endogenous OS, mainly due to their chemical stability and ubiquitousness in tissues and body fluids [21–23]. Elevated levels of plasma or urinary F_2_-IsoPs have been reported in several diseases [24–26]. For a correct O.S. damage quantification, Halliwell has suggested to use a combination of blood IsoPs and urinary IsoP metabolites determinations [27]. The 15-F_2t_-IsoP is the most represented isomer among the F_2_-IsoPs and is also referred to as 8-iso-prostaglandin F_2*α*_ [28]. 

In addition to F_2_-IsoPs with F_2_-ring, a variety of IsoPs with different ring structures, E_2_-ring and D_2_-ring, have been so far identified. Central in the pathway of formation of IsoPs are PGH_2_-like endoperoxides. Just as cyclooxygenase-derived PGH_2_ are rearranged to PGD_2_ and PGE_2_, the H_2_-IsoP endoperoxides can be reduced to form F_2_-ring IsoPs [29] but can also undergo rearrangements to form E_2_- and D_2_-IsoPs. Biological effects for E_2_- and D_2_-IsoPs have been described [30]. Subsequent to E_2_- and D_2_-IsoPs dehydration [29], cyclopentenone IsoPs can be formed [31]; cyclopentenone IsoPs, A_2_/J_2_-IsoPs, are highly reactive; *α*,*β*-unsaturated carbonyl moieties are highly susceptible to Michael addition reactions [32, 33]. In particular, one of the A_2_-IsoPs, 15-A_2t_-IsoP is primarily metabolized by these cells via conjugation to glutathione [34].

### 6.2. F_4_-Neuroprostanes

A series of F_2_-IsoP-like molecules named F_4_-neuroprostanes (F_4_-NeuroPs) originate from the free radical catalyzed peroxidation of DHA, an essential constituent of nervous tissue, highly enriched in neurons, and highly susceptible to oxidation [35]. Quantification of these compounds appears to provide a very sensitive index of oxidative neuronal injury in contrast to the IsoPs, since the amounts of F_4_-NeuroPs formed from DHA oxidation exceed the levels of F_2_-IsoPs generated from AA by 3.4-fold [36]. Given the well-known role for free radicals in the pathogenesis of a number of neurodegenerative diseases, that is, Alzheimer's disease, Parkinson's disease, Huntington's disease, and amyotrophic lateral sclerosis [20, 29, 37], the quantification of F_4_-NeuroPs appears to be a tool to evaluate a brain oxidative injury.

In experimental models of neurodegenerative phenomenon, F_4_-NeuroPs appear to be not related to excitotoxicity and epilepsy [38, 39], and a decrease of F_4_-NeuroPs levels, subsequent to treatment with antioxidant, was observed [39].

Furthermore, an increase of F_4_-NeuroPs has been reported in a neonatal hypoxic-ischemic encephalopathy [40]. The hypoxic-ischemia determines, in association with brain damage, formation of F_4_-NeuroPs, as well as F_2_-IsoPs. As F_4_-NeuroP levels are particularly elevated in ischemic areas, F_4_-NeuroPs may represent a specific marker of ischemic damage.

In Alzheimer's or Parkinson's diseases, F_4_-NeuroPs, as well as F_2_-IsoPs, are markedly increased in brain tissue and cerebrospinal fluid [41]. In particular, the F_4_-NeuroP levels were significantly higher as compared to those of F_2_-IsoPs, in the brain regions affected by the disease [42, 43]. Increased levels of F_4_-NeuroPs have been also reported in the cerebrospinal fluid of patients with subarachnoid hemorrhage from aneurysm. The generation of F_4_-NeuroPs in subarachnoid aneurysm hemorrhage and traumatic brain injury [44, 45] appears to be the consequence of a catastrophic central nervous system injury, and it can be considered an useful indicator of the pathological event. Regards to F_4_-NeuroPs determination, some researchers have used the chemically synthesized 17-F_4c_-NeuroP isomer [46], although interference with F_2_-dihomo-isoprostanes (F_2_-dihomo-IsoPs) cannot be ruled out [44].

### 6.3. F_2_-Dihomo-Isoprostanes

F_2_-dihomo-IsoPs are specific markers for free radical-induced AdA peroxidation and have been characterized as potential markers of free radical damage to myelin in human brain [47]. To date, clinical applications for F_2_-dihomo-IsoPs are few and studies are reported for white brain matter [47], cerebrospinal fluid [44], and plasma [48]. For the first time, our studies have shown the possibility of F_2_-dihomo-IsoP evaluation in plasma.

## 7. IsoPs as Mediators of Disease

15-F_2t_-IsoP is previously also indicated as 8-epi-PGF2*α* or 8-iso-PGF2*α*, as it is one of the most abundantly F_2_-IsoP isomer produced *in vivo* and may exhibit biological activity [24].

F_2_-IsoPs seem activate receptors analogous or identical to those for the thromboxane A_2_ (TxA_2_) and induce platelet aggregation [49] and vasoconstriction of renal glomerular arterioles [50, 51]. Via activation of the TxA_2_ receptors, IsoPs inhibit angiogenesis [52]. Furthermore, stimulation of DNA synthesis and cell proliferation for F_2_-IsoPs on muscle vascular cells [51] and endothelial cells [53] is known, as well as the role of F_2_-IsoPs in the pulmonary pathophysiology [54]. In streptozotocin-induced diabetes, F_2_-IsoPs appear to mediate an increase of the transforming growth factor-*β*1 (TGF-*β*1) [55], while the 5-F_2t_-IsoPs and 5-epi-5-F_2t_-IsoPs regulate the [³H]d-aspartate release in isolated bovine retina [56].

The complex F_2_-IsoPs bioactivity has been summarized in recent reviews [57–59].

## 8. The Role of Chemical Synthesis in the Exploration of Lipid Mediators 

Oxidative stress is evolved in neurodegeneration of grey matter (Alzheimer's diseases) [60] and white matter (multiple sclerosis or Rett syndrome).

As their isomers F_2_-IsoPs are known as the “gold standard” for systemic O.S. [61] and for their biological activities [62, 63], F_4_-NeuroPs and F_2_-dihomo-IsoPs are potential biomarkers in specific pathologies such as AD or RTT and may as well have biological activities. 

In order to demonstrate theses various activities of PUFAs oxygenated metabolites, chemical synthesis is a need and chemists are the link between biochemists and biologists.

**Scheme 1:**
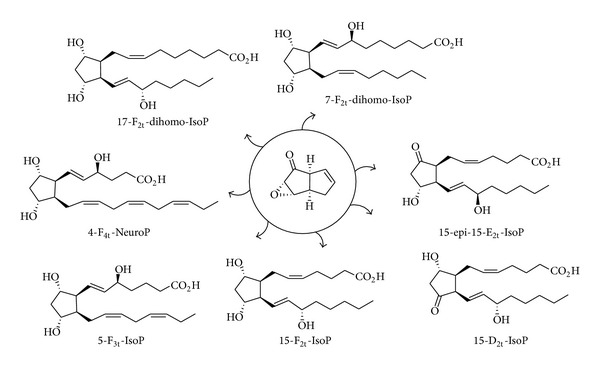


Thus, since the discovery of F_2_-IsoPs, chemists developed strategies to access to IsoPs [64–66] as well as NeuroPs [67, 68], dihomo-IsoPs [69, 70]. Among them, our laboratory, specialized in total synthesis of lipids metabolites (leukotrienes, isoprostanes, and resolvins), developed during the past twenty years three strategies allowing the access to F-type IsoPs [69, 71, 72] as well as E-type [69, 73, 74], D-type [74], and A-type (Bultel-Poncé et al., unpublished results). Those strategies permitted the syntheses of different series of oxygenated metabolites derived from *α*-linolenic acid (ALA), AA, DHA, EPA, and AdA (Scheme 1).

With those chemically pure metabolites in hands, biologists and clinicians have shown biomarkers activities [48] as well as biological activities [75, 76] of those oxidative stress-derived metabolites.

## 9. Rett Syndrome: A Genetic Model of Autism Spectrum Disorder

RTT (OMIM ID: 312750) occurs with a frequency of up to 1/10,000 live female births. Causative mutations in the X-linked methyl-CpG binding protein 2 gene (*MECP2*) are detectable in up to 95% of cases, although a wide genetical and phenotypical heterogeneity is well established [77]. Approximately 80% of RTT clinical cases show the so-called “typical” clinical picture; after an apparently normal development for 6–18 months, RTT girls lose their acquired cognitive, social, and motor skills in a typical 4-stage neurological regression and develop autistic behavior accompanied by stereotypic hand movements [78]. Autistic features are typically transient in RTT.

In addition to typical RTT, it has been recognized that some individuals present with many, but do not necessarily all, of the features of the disorder. New guidelines for the diagnosis of specific “variant” or “atypical” forms of RTT have been developed to identify the preserved speech, early seizure, and the congenital variant [79].

Although the genetic mechanisms of disease have been extraordinary explored in details in RTT, to date the biological mechanisms linking the gene mutation to the phenotypic expression of the disease including its wide heterogeneity are yet to be clarified. Several explanations have been proposed so far, including a key role of *MECP2* in the neuronal maturation [80], maintenance of astroglia, immune dysfunction [81], and neurotransmission pathway abnormality [82]. However, recent discoveries, mainly by our team, concerning the emerging role of alteration of redox homeostasis offer an alternative explanations which is not mutually exclusive with the others previously proposed. Nevertheless, the whole history of RTT is cluttered with several apparently firm points that have been subsequently changed. Interestingly, one of the most firm points to date is that RTT is caused by a single-gene mutation either *MECP2* or other more rarely affected (i.e., *CDKL5* or *FOXG1 *genes) [79]. Recently the analysis of the full exome sequencing in two pairs of affected sisters, each with identical *MECP2* gene mutation but discordant phenotype, indicates that several hundreds of gene mutations appear to be associated with the *MECP2* gene mutation and therefore are to be considered previously unknown disease modifier [83]. 

Currently, no effective pharmacological therapies for RTT exist that can either halt progression, or reverse the neurological and cognitive abnormalities.

## 10. Lipid Peroxidation and Rett Syndrome

Mounting evidence indicates an emerging role for O.S. in genetically determined diseases [84]. Furthermore, the role of the redox alteration in the pathogenesis of the autism spectrum disorder is under debate [85, 86].

In 2001 indirect evidence for excessive lipid peroxidation, leading to increased plasma malondialdehyde levels, has been reported in RTT patients [87]. However, after that isolated report and before our subsequent series of specific studies in the field, the lipid oxidative damage in RTT had not been further investigated. Our studies were focused on the identification of different classes of IsoPs increased in RTT, thus allowing an inference on the individual oxidized fatty acids precursors relevant to the disease. On the other hand, our reports of increased 4-HNE PAs levels in RTT patients while further supporting the evidence of a lipid damage with formation of the aldehyde 4-HNE also detect the presence of a coexisting protein damage due to the formation of the adducts.

Therefore, our findings in RTT, a rare cause of genetically determined autism spectrum disorder, indicate that this pervasive development disorder can be considered a unique human model for chronic O.S. and could be considered a valuable testing ground for the link between lipid peroxidation byproducts and the mediation of disease processes.

### 10.1. Isoprostanes and Neuroprostanes in RTT

Our findings [48, 88–90] indicate that typical RTT is characterized by markedly increased levels of IsoPs deriving from the nonenzymatic oxidation of AA, DHA, and AdA at every clinical stage of the disease. IsoPs and NeuroPs levels appear to be closely interrelated to the RTT clinical presentation, suggesting that these lipid oxidation products could mediate the pathogenetic mechanisms underlying the syndrome. 

Extremely high (i.e., two orders of magnitude) plasma levels of F_2_-dihomo-IsoPs are detectable in RTT girls in stage I of the disease. AdA, whatever the actual origin (brain white matter, adrenal gland, or kidney), is the PUFA that goes through the greatest degree of oxidation during the earliest stage of the typical form of the disease. An insult to AdA and the clinical onset of neuroregression occur at the same time [48]. Thus, during the first two years of the natural evolution of the disease, the peroxidation of AdA, a critical component of myelin [47] in the primate brain, is involved.

Oxidation of the AA appears to be another essential component in the pathogenesis of the first two stages of typical RTT, as can be deduced from the significantly high F_2_-IsoPs in the early stages as compared with the late natural progression of classic RTT. Nevertheless, F_2_-IsoPs increase not with the same marked raise of the F_2_-dihomo-IsoPs. Thus, F_2_-dihomo-IsoPs are the prominent lipid peroxidation end-product detectable at this stage of the disease. In RTT girls, plasma F_2_-IsoPs are always above the physiological range, and thus, considering the half-life of these prostanoids, lipid peroxidation is continuously carried out in the syndrome. Due to the systemic distribution of AA, free plasma F_2_-IsoPs can be considered an index of generalized lipid peroxidation, while in RTT, a specific site of peroxidation events has been identified in the erythrocyte membrane. In fact, esterified F_2_-IsoPs are increased in RTT erythrocyte membrane and their levels are correlated to altered red blood cells shape [91].

Patients with typical RTT had significantly higher F_2_-IsoPs than those with atypical phenotype and are correlated to RTT clinical severity, as well as F_4_-NeuroPs. In particular, F_4_-NeuroPs are related to clinical severity markers, including early regression, severe head growth deceleration, major motor impairment, hand use loss, and seizures. In our experience, *MECP2 *gene mutations located in critical regions that carry higher phenotype severity usually show a more severely shifted O.S. imbalance [89, 90]. Moreover, the demonstrated link between F_4_-NeuroPs and *MECP2* genotype-phenotype correlation suggests that the degree of MeCP2 protein dysfunction is directly proportional to the O.S.-mediated neuronal damage, explaining ~90% of the expressed phenotype variability [90]. Also plasma F_2_-IsoPs concentrations are significantly related to *MECP2* genotype; anyway, the strength of the relationship between plasma F_2_-IsoPs and MeCP2 phenotype appears to be far weaker than that between plasma F_4_-NeuroPs and MeCP2 phenotype [89, 90]. As F_4_-NeuroPs plasma levels mirror neurological severity, these molecules may provide evidence on neuronal damage, also considering the specific distribution of DHA in membrane neurons.

### 10.2. 4-HNE and RTT

In our recent work, we have shown that the levels of 4-HNE PAs change during the clinical progression of typical RTT [92]. Plasma 4-HNE PAs increase between stage I and stage II and to less extent in the following stages (i.e., III and IV). 

Impairment of the GSH system and other detoxification enzymes involved in aldehyde metabolism or defect/dysfunction in the ubiquitin proteasome system, reported in the autism spectrum disorder [93–95], could be involved in the accumulation, in the early stages of RTT, of several 4-HNE plasma protein adducts. This, as a consequence, could play a role in the severe clinical features observed in the later stages of the disease. 

As for the IsoPs, a relationship between 4-HNE PAs and phenotypical RTT presentation has been reported. In particular, with reference to the atypical RTT clinical presentation, *CDKL5*-related RTT patients have a significant increase in 4-HNE PAs levels, and on the contrary, *FOXG1*-related RTT patients are not different from the controls [92]. Although the possible cause for the observed difference in *FOXG1*-related RTT is not clear, the 4-HNE PAs, as well as the IsoPs, appear to be directly involved in the RTT pathogenesis. To date, it is not clear how the gene mutations can induce and increase O.S. in RTT patients. It is possible to speculate that it could be an indirect mechanism that might involve mitochondria respiration, modifying therefore the redox state of the cells. 

It should be pointed out that the presence of 4-HNE PAs in plasma of RTT patients suggests two events. First, it is indicative of a generalized lipid peroxidation, indicating the occurrence of PUFAs oxidation in various organs and/or tissues. Second, by covalent modification of proteins, 4-HNE is able to cause long-lasting biological consequences. Therefore, plasma proteins can be considered a target of increased O.S. status in RTT. Hence, it is possible to speculate that 4-HNE PAs can contribute to the pathophysiology of RTT, both in the development and in the progression and complications of the disease. We can suggest that 4HNE PAs represent a potential biomarker of RTT as well as disease severity. 

In future, to better understand the clinical consequences that the modified plasma proteins have on RTT patients, further studies are needed. It could be of extreme importance to be able to identifying the target proteins modified by 4-HNE in the plasma, as recently shown for mild cognitive impairment and Alzheimer's disease [96, 97]. 

A close relationship between levels of circulating lipid peroxidation markers in RTT patients and presence of the symptom is not a causative proofs but strongly supports the concept that lipid peroxidation plays a previously unrecognized key role in the pathogenesis of RTT due to the gene mutation. The increase of knowledge on nature of 4-HNE modified proteins in RTT, might lead to better prevention, diagnosis and treatments of the associated physiological processes altered in patients. 

Finally, the involvement of lipid peroxidation in RTT was also confirmed in our recent study, where the morphology of erythrocytes in typical RTT patients has been evaluated [91]. Emerging evidence indicates that O.S. imbalance and hypoxemia can lead to erythrocyte shape abnormalities in chronic pulmonary disease [98, 99], and now it has been well proved that chronic hypoxia, impaired pulmonary gas exchange, and increased O.S. are all present in typical RTT [88]. In fact, our data show the presence of erythrocytes altered shape (mainly leptocytes) and membrane oxidative damage (i.e., 4-HNE PAs) in patients with clinical diagnosis of typical RTT [91]. Consequently, monitoring of erythrocytes morphology can be an important new diagnostic and prognostic tool in this particular form of autism spectrum disorder, in which the lung seems to represent an unexpectedly key organ for the disease pathogenesis.

### 10.3. Lipid Peroxidation and *ω*-3 PUFAs Supplementation in RTT

**Figure 1:**
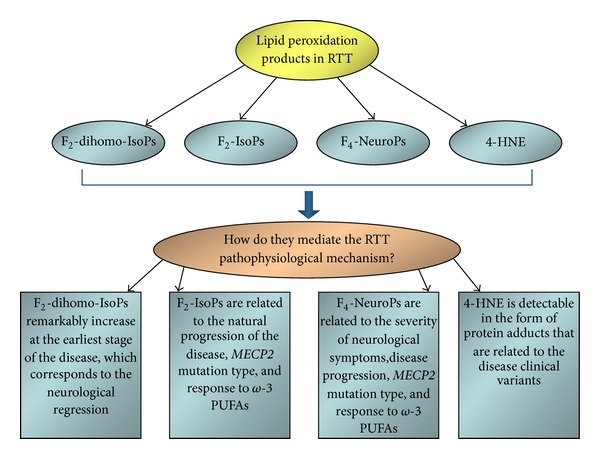
Different classes of isoprostanes (F_2_-dihomo-IsoPs, F_2_-IsoPs, and F_4_-NeuroPs), deriving from polyunsaturated fatty acids precursors (adrenic, arachidonic, and docosahexaenoic acids, resp.), and the 4-hydroxy-2-nonenal protein adducts are strictly related to the clinical manifestations and the natural history of Rett syndrome. The lipid peroxidation events and the disease pathogenic mechanisms are closely interrelated, as demonstrated by the dietary supplementation with *ω*-3 PUFAs. RTT: Rett syndrome; F_2_-dihomo-IsoPs: F_2_-dihomo-isoprostanes; F_2_-IsoPs: F_2_-isoprostanes; F_4_-NeuroPs: F_4_-neuroprostanes; 4-HNE: 4-hydroxy-2-nonenal; and PUFAs: polyunsaturated fatty acids.

Lipid peroxidation appears to be a peculiar characteristic of RTT, and the relationships between the described lipid peroxidation products and the clinical disease features are summarized in Figure 1.

A better comprehension of the lipid peroxidation involvement in the pathogenetic mechanisms of the disease derives from studies carried out with *ω*-3 PUFAs supplementation. Interestingly, the supplemented molecules are actually just the same category of molecules that undergo radical damage. Exogenous administration of *ω*-3 PUFAs, in disease stages I-IV, has been shown to moderately reduce clinical severity and significantly reduce the levels of IsoPs and 4-HNE PAs in RTT patients [89–91, 100]. Following *ω*-3 PUFAs supplementation, we might have expected an enhanced formation of the lipid oxidation products in plasma. Actually, the assumed fatty acids are not oxidized and the endogenous production is reduced. These results lead us to think that in RTT the lipid-oxidative damage is nonspecific, all fatty acids are not oxidatively damaged, but the oxidative insult regards specific biological targets.

As a consequence, these data, while confirming the involvement of lipid products in the intimate pathogenic mechanisms of the disease, indicate that the fatty acid oxidation is related to the clinical severity of the disease and is a reversible process. 

## 11. Conclusion

Lipid peroxidation end-products appear to be associated with the modulation of RTT disease severity, thus suggesting their likely role as mediators. The identification of the involved classes of molecules (including metabolites) in the disease, the evaluation of their relationship with the disease natural history, and the chance to determine the effects of treatments (i.e., exogenous PUFAs supplementation) and of gene manipulation (i.e., reactivation of *Mecp2* null mice) offer a unique setting for physicians, biologists, and chemists to explore the borders of the lipid mediators concept.
